# Supplementary figures and images for: Development of a Chicken Immunoglobulin Heavy Chain Variable Region (VH) Single-Domain Antibody (sdAb) Against Calsequestrin (CSQ) and Its Application
Source: Antibodies (Basel). 2025 Sep 19;14(3):80. doi: 10.3390/antib14030080 (PMC12452619; doi:10.3390/antib14030080)

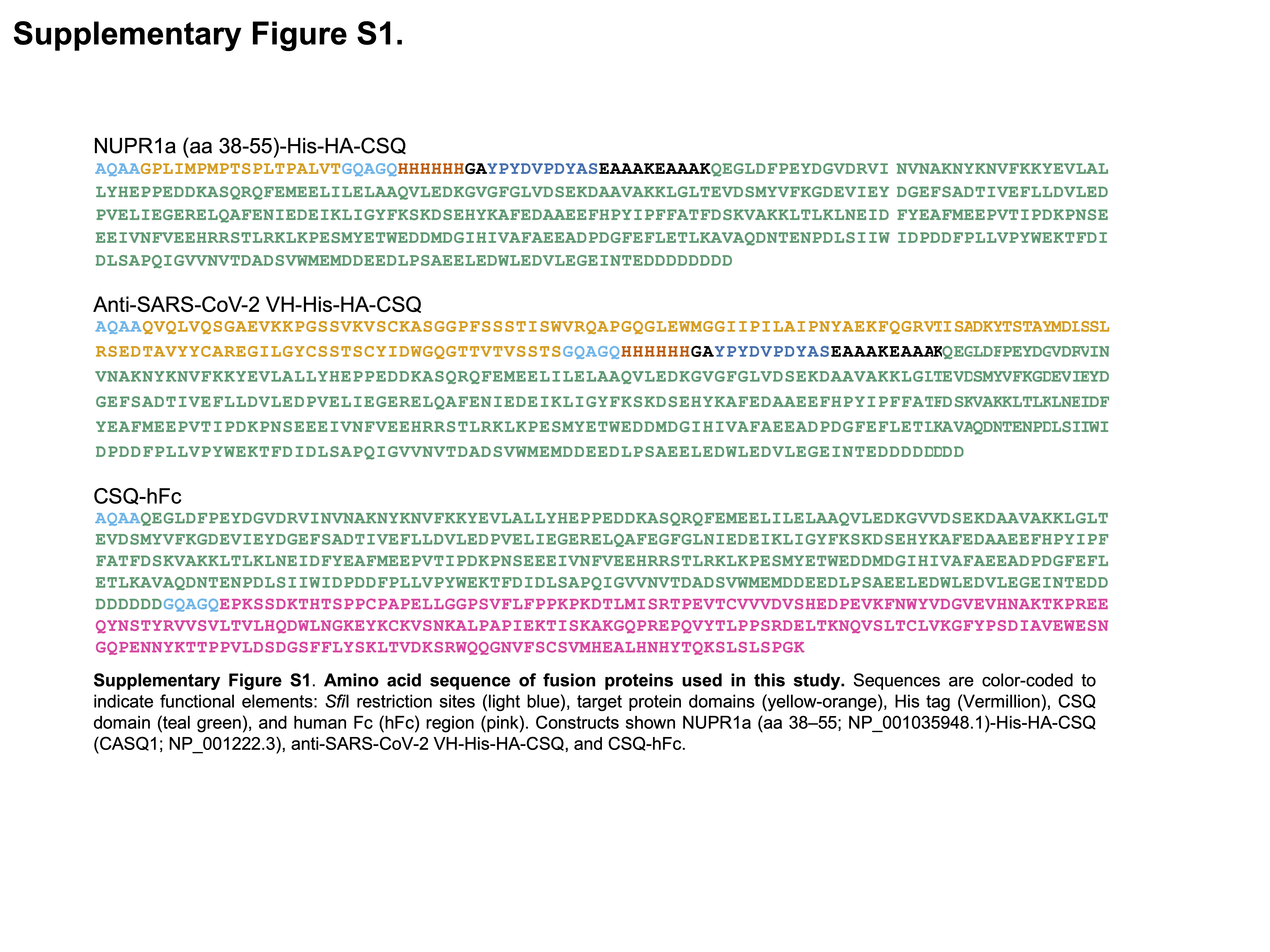

Supplement: Supplementary file 1 [file antibodies-14-00080-s001.zip › antibodies-3846980-supplementary/supplementary Fig. S1.png]

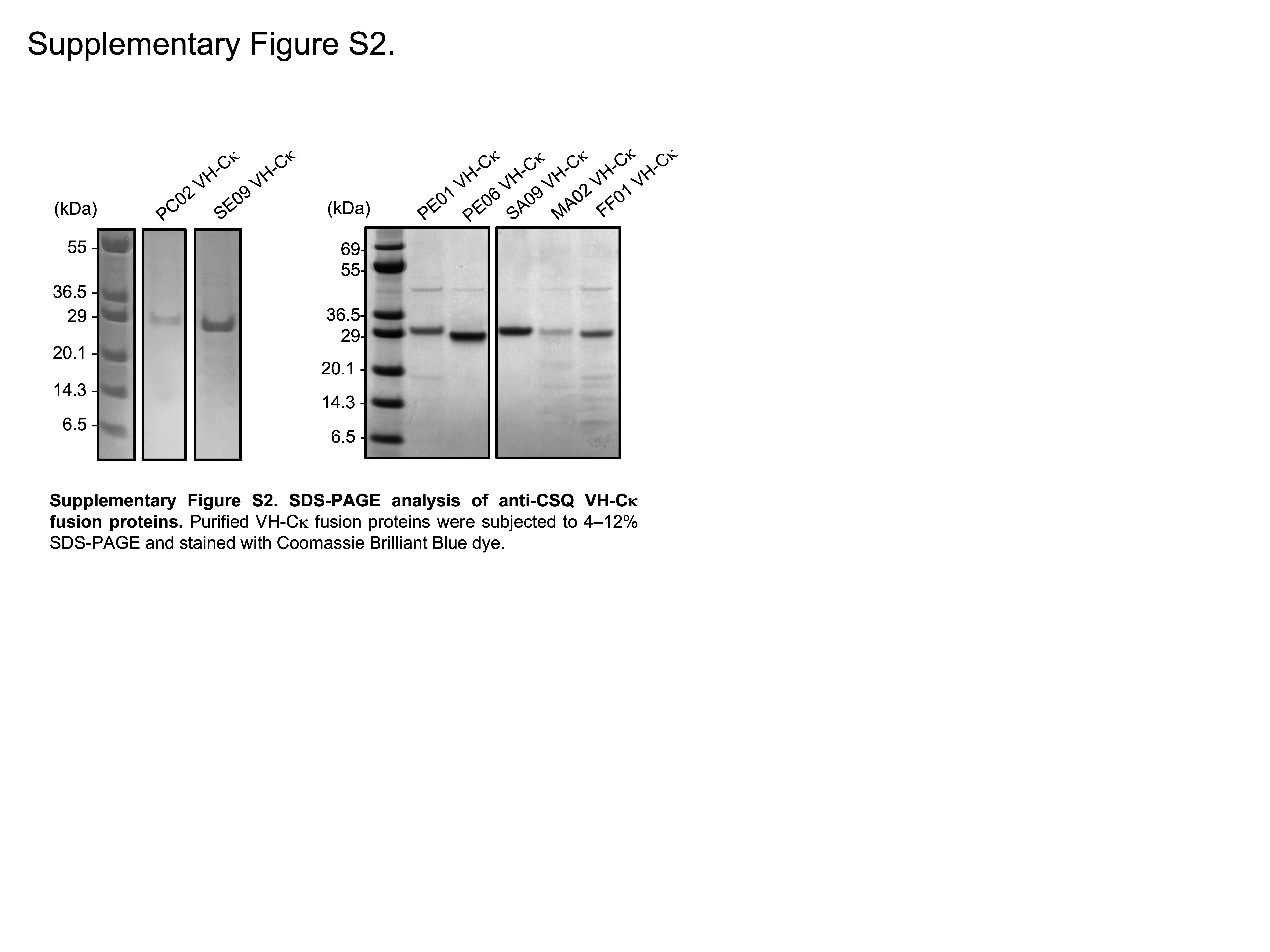

Supplement: Supplementary file 1 [file antibodies-14-00080-s001.zip › antibodies-3846980-supplementary/supplementary Fig. S2.png]

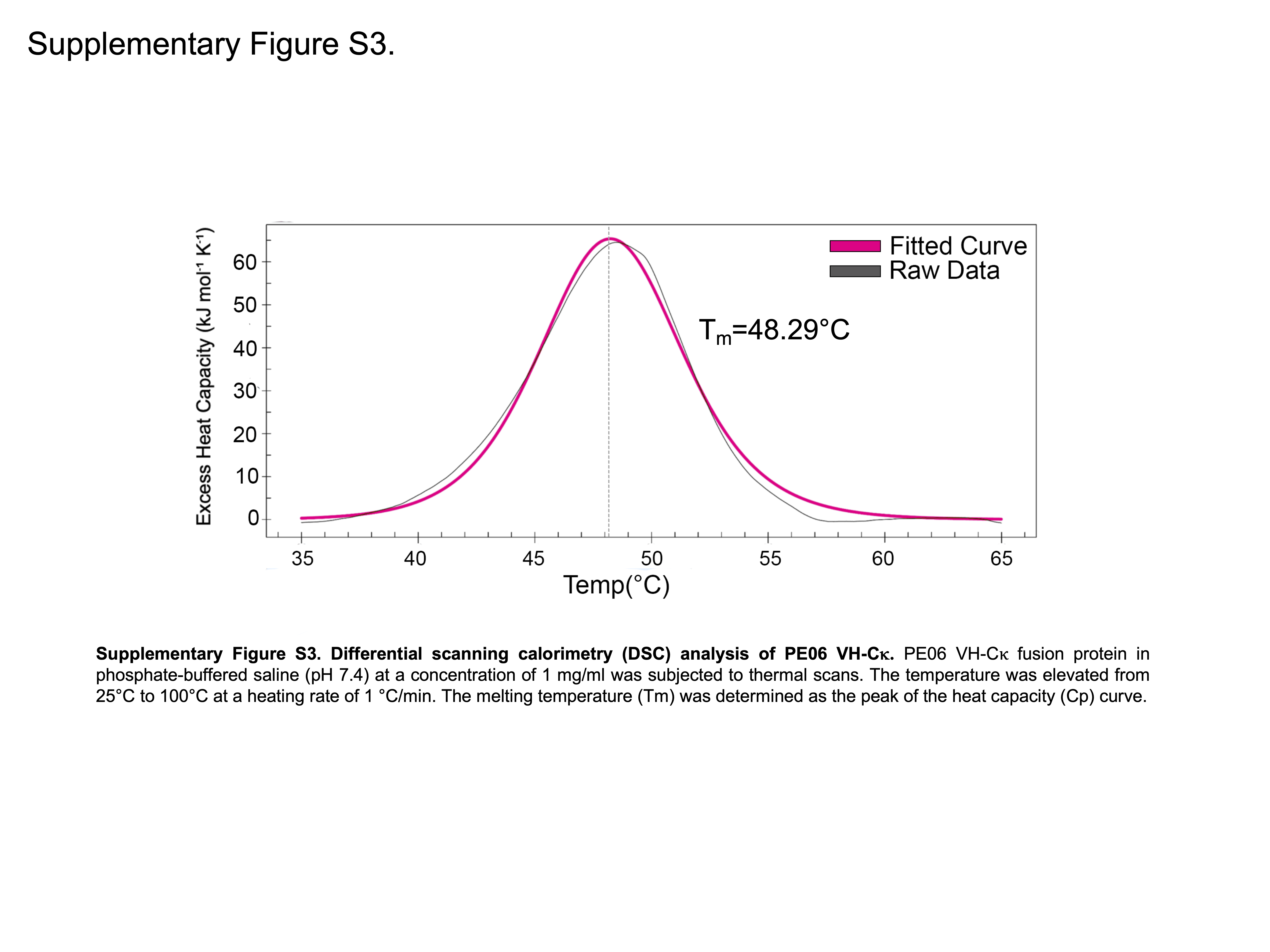

Supplement: Supplementary file 1 [file antibodies-14-00080-s001.zip › antibodies-3846980-supplementary/supplementary Fig. S3.png]

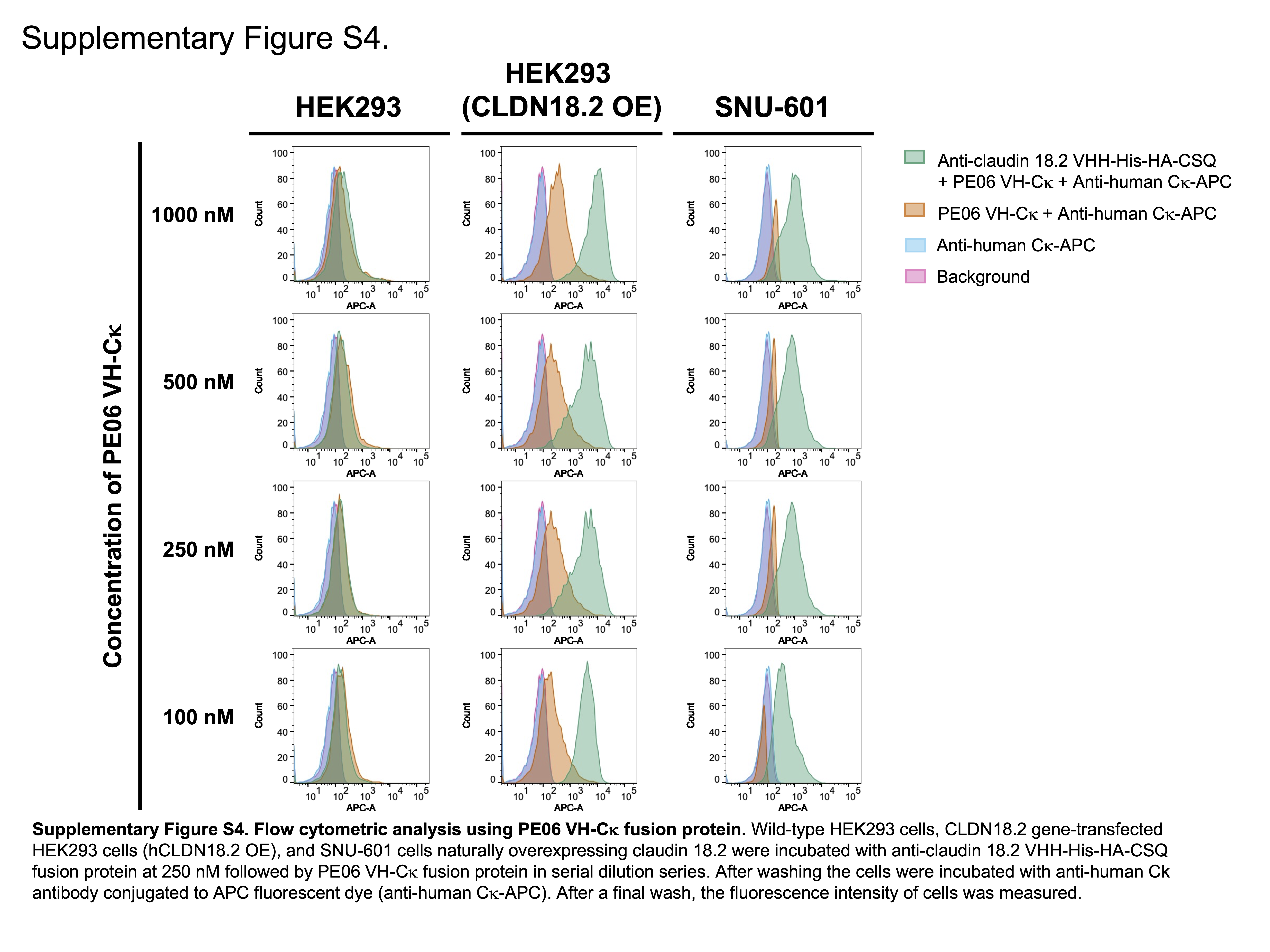

Supplement: Supplementary file 1 [file antibodies-14-00080-s001.zip › antibodies-3846980-supplementary/supplementary Fig. S4.png]

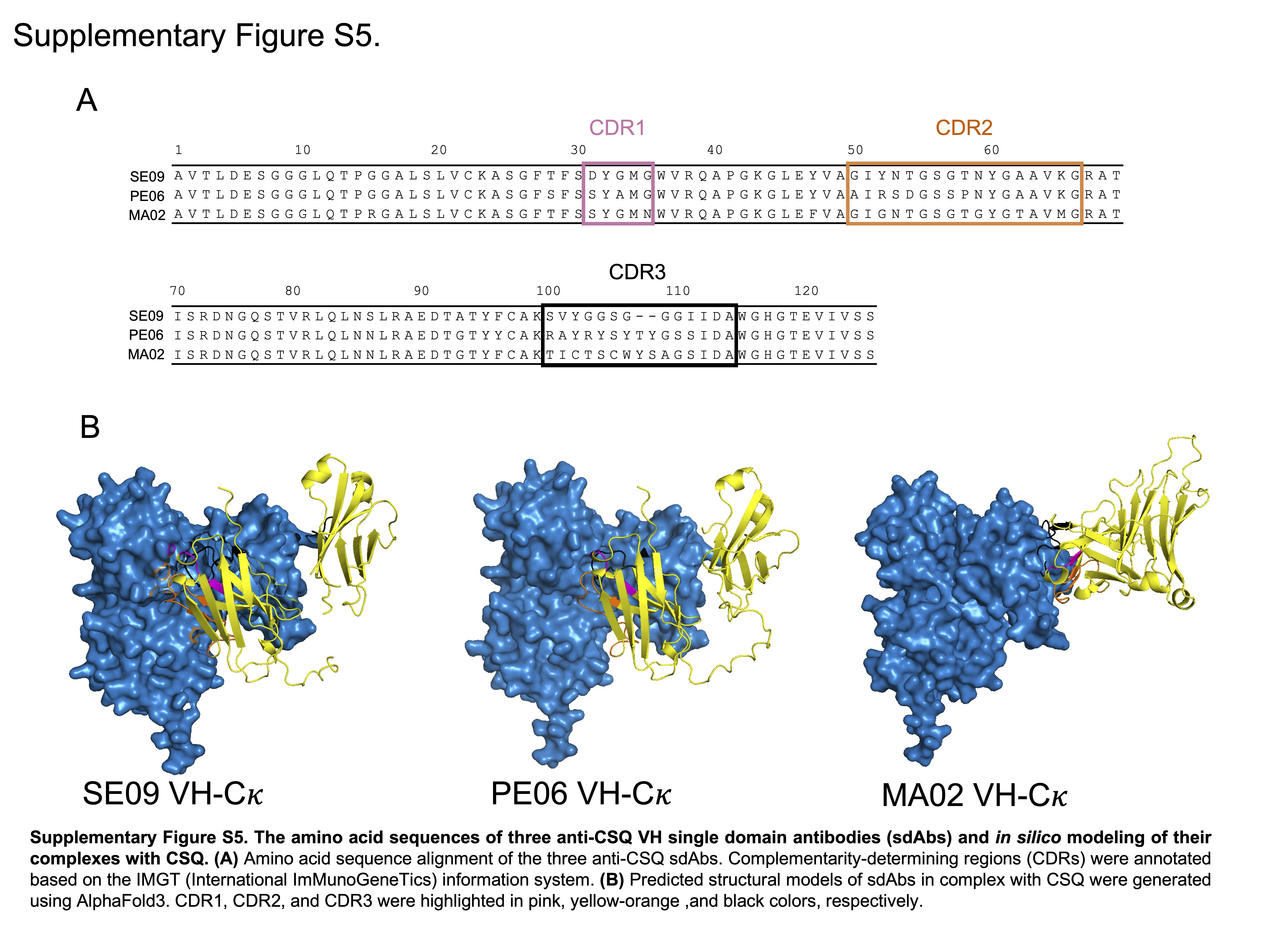

Supplement: Supplementary file 1 [file antibodies-14-00080-s001.zip › antibodies-3846980-supplementary/supplementary Fig. S5.png]

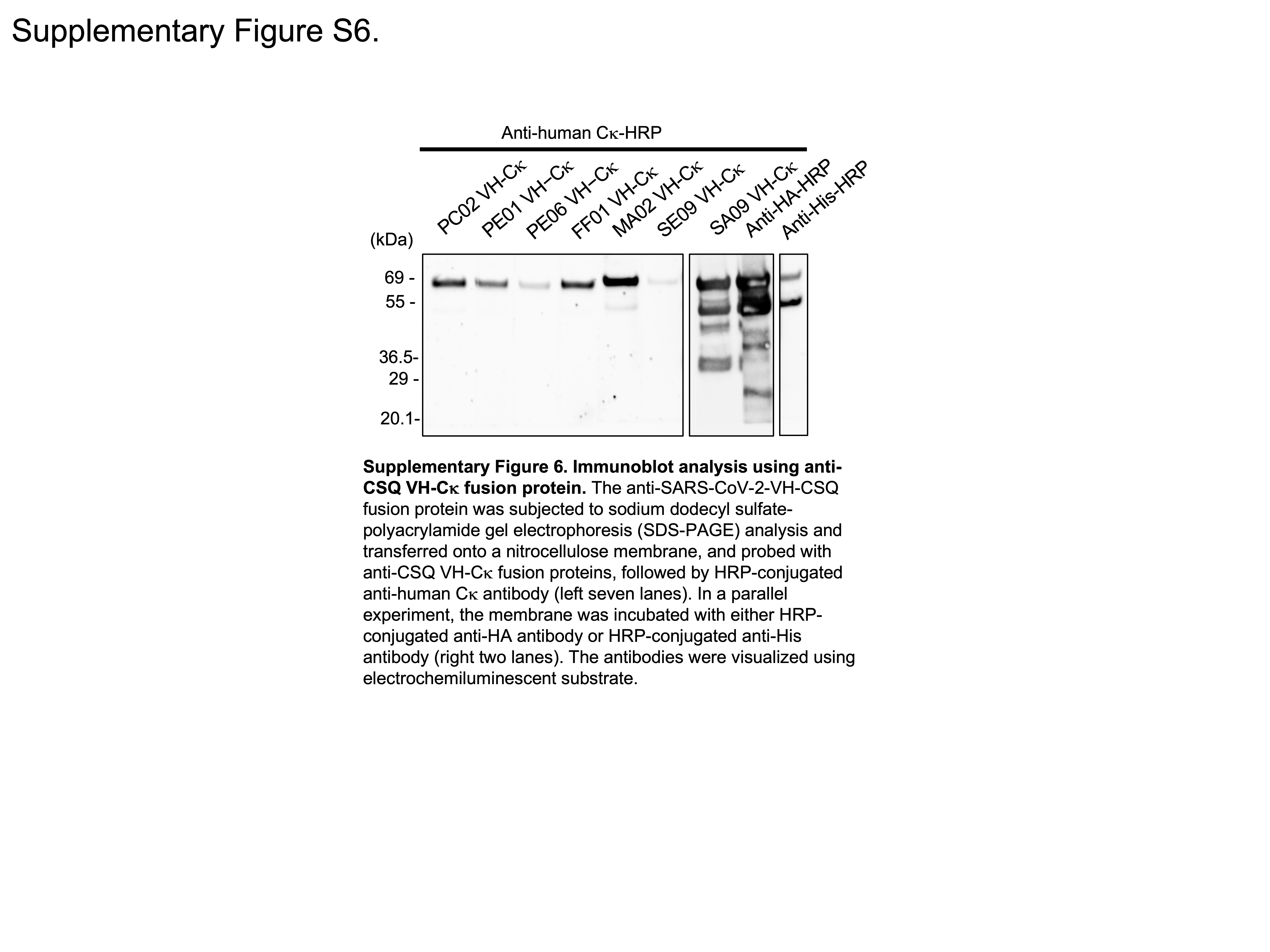

Supplement: Supplementary file 1 [file antibodies-14-00080-s001.zip › antibodies-3846980-supplementary/Supplementary Fig. S6.png]

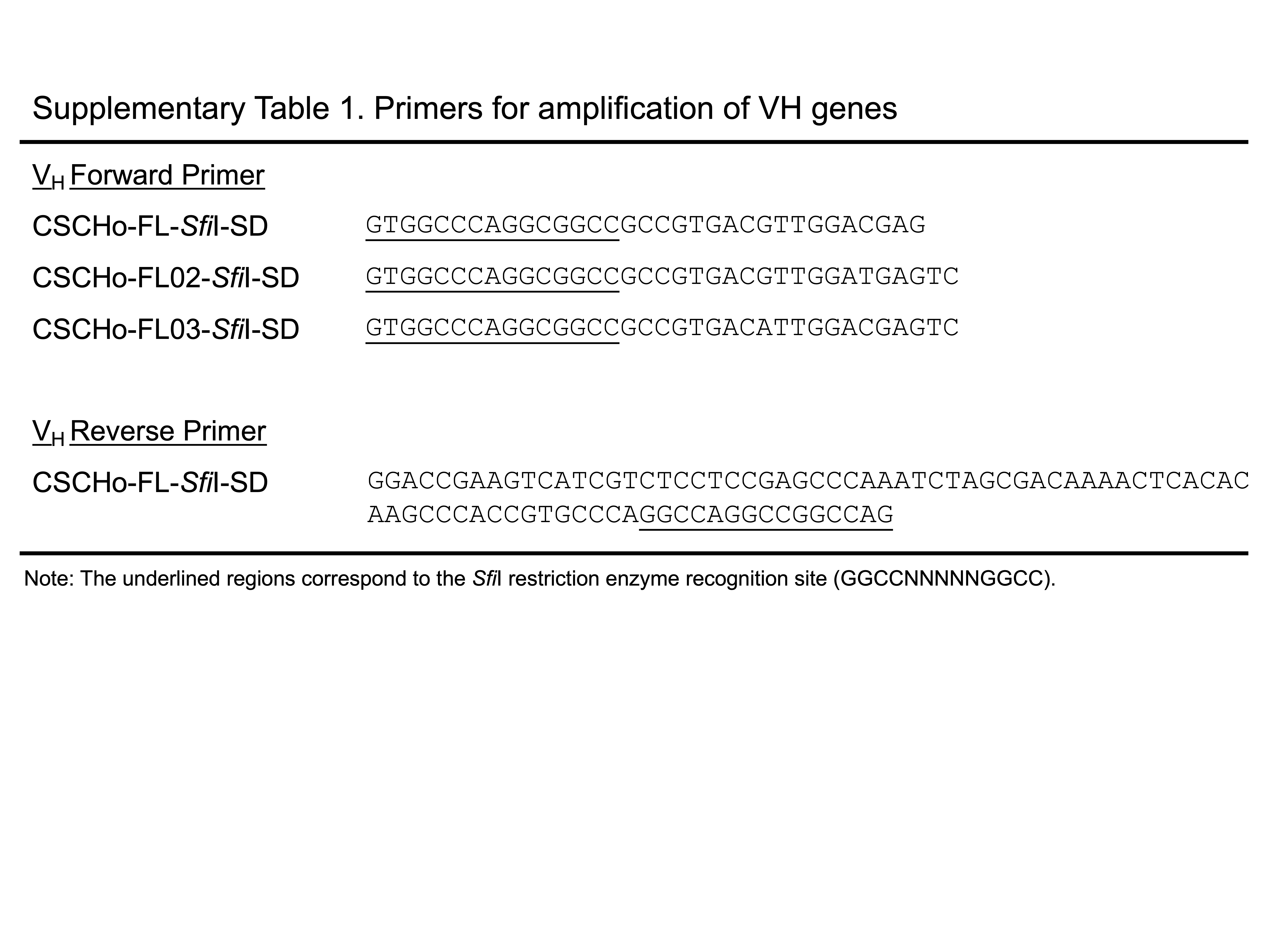

Supplement: Supplementary file 1 [file antibodies-14-00080-s001.zip › antibodies-3846980-supplementary/Supplementary Table. S1.png]
